# Associated bacteria of a pine sawyer beetle confer resistance to entomopathogenic fungi via fungal growth inhibition

**DOI:** 10.1186/s40793-022-00443-z

**Published:** 2022-09-09

**Authors:** Jundan Deng, Weikang Xu, Guochang Lv, Hang Yuan, Qing-He Zhang, Jacob D. Wickham, Letian Xu, Longwa Zhang

**Affiliations:** 1 Anhui Provincial Key Laboratory of Microbial Control, Anhui Agricultural University, Hefei, 230036 China; 2 State Key Laboratory of Biocatalysis and Enzyme Engineering, School of Life Sciences, Hubei University, Wuhan, 430062 China; 3 Sterling International, Inc., Spokane, WA 99216 USA; 4 A.N. Severtsov Institute of Ecology and Evolution, Russian Academy of Sciences, 33 Leninsky Prospect, Moscow, Russia 119071

**Keywords:** *Monochamus alternatus*, *Beauveria bassiana*, Associated bacteria, Inhibition

## Abstract

**Background:**

The entomopathogenic *Beauveria bassiana* is a popular fungus used to control the Japanese pine sawyer, *Monochamus alternatus* Hope, the key vector of pine wood nematode (*Bursaphelenchus xylophilus*) that is the causal agent of pine wilt disease, resulting in devastating losses of pines in China and Portugal. However, recent studies have demonstrated that some insect-associated bacteria might decrease fungal toxicity and further undermine its biological control efficacy against *M. alternatus*. Thus, it is of great significance to uncover whether and how associated bacteria of *M. alternatus* become involved in the infection process of *B. bassiana.*

**Results:**

Here, we show that axenic *M. alternatus* larvae died significantly faster than non-axenic larvae infected by four increasing concentrations of *B. bassiana* spores (Log-rank test, *P* < 0.001). The infection of *B. bassiana* significantly changed the richness and structure of the beetle-associated bacterial community both on the cuticle and in the guts of *M. alternatus*; meanwhile, the abundance of *Pseudomonas* and *Serratia* bacteria were significantly enriched as shown by qPCR. Furthermore, these two bacteria genera showed a strong inhibitory activity against *B. bassiana* (One-way ANOVA, *P* < 0.001) by reducing the fungal conidial germination and growth rather than regulating host immunity.

**Conclusions:**

This study highlights the role of insect-associated bacteria in the interaction between pest insects and entomopathogenic fungi, which should be taken into consideration when developing microbial-based pest control strategies.

**Supplementary Information:**

The online version contains supplementary material available at 10.1186/s40793-022-00443-z.

## Background

The pine sawyer beetle *Monochamus alternatus* Hope (Coleoptera, Cerambycidae) is a key insect vector of the pinewood nematode, *Bursaphelenchus xylophilus* (Steiner & Buhrer) Nickle, a destructive forest pest which has caused widespread and destructive pine wilt disease (PWD) throughout Asia and Europe [1–3]. The pinewood nematode, native to North America (USA and Canada), was first discovered in China in 1982 [4, 5]. Since then, it has spread to 15 provinces and caused tremendous environmental and economic damage (SFA.2020) due to its capability to kill healthy pine trees. A key strategy to prevent this nematode from spreading and damaging forests is to control the spread of its vector *M. alternatus* [6].

*Beauveria bassiana* has been used as an effective and practical insect biocontrol agent due to its strong pathogenicity and high adaptability for many years [7]. Although more evidence is still needed regarding *B. bassiana*’s high genetic diversity that in turn is often associated with varied virulence, the pathogenicity of *B. bassiana* against *M. alternatus* has already been well-proven in previous studies. For example, Zhang et al. [8] reported that all pupae of *M. alternatus* died after being infected by *B. bassiana* within 10 days. However, in addition to varied virulence of different *B. bassiana* strains, the pathogenicity of the fungus could be easily influenced by a series of biological factors, such as insect innate immunity (cellular and humoral) [9] and associated bacteria [10]. Managers may be required to re-apply the bioinsecticides multiple times or increase their concentrations dramatically to reach desired levels of control efficacy [11].

Among the aforementioned factors, insect-associated bacteria, thriving on the insect’s cuticular surface, within the gut, or in specialized insect microbe hosting structures, present a most elusive role in the process of entomopathogenic fungal infection [10, 12]. Previously, we discovered that *Dendroctonus valens* LeConte infection with *B. bassiana* caused a dysbiosis of gut microbiota, and the overgrowth of the bacterium *Erwinia* sp. in the gut, which accelerated the infection of entomopathogenic fungi [13]. Similar results were also reported in mosquitoes [14]. In addition to a synergistic effect between fungal and insect associated bacteria interactions, antagonistic effects between these two agents were also widely reported. The insect-associated bacteria play an important role as an indispensable “defense organ” when the host insect was infected or parasitized by pathogens [15]. For example, five aphid clones that possess endosymbiont *Regiella insecticola* are more resistant to the entomophthorales fungus *Pandora (Erynia) neoaphidis*, and *Regiella* reduced sporulation frequency of fungus [16]. Associated bacteria of *Apis mellifera*, *Blattella germanica, Galleria mellonella* and *Philanthus triangulum* have also been demonstrated to exert antifungal activity similar to the above study [17–20]. Recently, *M. alternatus* was found to harbor a large number of associated bacteria [21, 22], but their role in the infection process of *B. bassiana* remains unclear.

In this work, we first determined the role of associated bacteria of *M. alternatus* in the fungal infection process by evaluating the susceptibility of non-axenic and axenic larvae to increasing concentrations of *B. bassiana* spores*.* We then analyzed how the fungus influenced gut- and cuticle-associated microbiota of *M. alternatus* using high-throughput sequencing platform and qPCR*.* The antifungal activity of the enriched bacteria during the infection process was further tested and confirmed by examining their inhibitory effect on the growth and germination of *B. bassiana* spores in vitro. Finally, the immunity of axenic and non-axenic larvae against *B. bassiana* was evaluated. Our results suggest that bacteria associated with *M. alternatus* may serve as an extra line of defense against the *B. bassiana* infection.

## Methods

### Insect maintenance and antibiotic treatment

In September 2019 and 2020, the third instar larvae of *M. alternatus* were collected from the beetle-infested pine trees (*Pinus massoniana*) in the Jiuhua Mountain (30°5′N, 117°8′E), Chizhou city, Anhui province, China. Larvae were placed in 6-cm disposable petri dishes with artificial medium and fed with an artificial diet (made of 15 g agar mixed with 210 g powder of pine, then added to 1 L boiled distilled water) [23], at 25 ± 1 °C. The artificial diet was refreshed each day.

For antibiotic treatment, *M. alternatus* larvae were washed with 75% alcohol for 30 s and rinsed with sterile water, then transferred on artificial diet containing streptomycin sulfate, ampicillin sodium salt, tetracycline HCl and nystatin each at 1 mg/mL, respectively. Germ-free (axenic) larvae were obtained after rearing for 4 days.

To determine whether gut and cuticle microbes were eliminated completely, third instar larvae were dissected under sterile conditions and gut and cuticle samples were collected in 1.5 mL DNase/RNase-free centrifuge tube with 200 μL 10% phosphatic buffer solution. Three gut samples from *M. alternatus* with antibiotic treatment were assigned as axenic, and three from untreated larvae were non-axenic. The suspension was serially diluted 1000 times with sterile water, plated onto Luria–Bertani media (LB) agar plates and tryptic soy agar (TSA). In addition, we also measured gut bacteria by 16s rRNA qPCR with 16sF (5′-TCCTACGGGAGGCAGCAGT-3′) and 16sR (5′-GGACTACCAGGGTATCTAATCCTGTT-3′) primers (qPCR method is specifically described below). Results showed we successfully obtained axenic insects (Additional file 1: Fig. S1).

### Survival analysis

To obtain *B. bassiana* (Bb3275) blastospores, Sabouraud’s Dextrose Agar (1% polypeptone, 4% glucose, 1.5% agar) supplemented with 1% yeast extract (SDAY) was inoculated with the fungus and incubated at 26 °C for 14 days. Then, conidial suspensions were dispersed in a sterile solution of 0.05% (v/v) Tween 80 and increasing concentrations of 1 × 10^6^–1 × 10^9^ conidia mL^−1^ were determined by direct counting using a hemocytometer.

To infect *M. alternatus*, 25 axenic and 25 nonaxenic *M. alternatus* larvae were dipped for 10 s into *B. bassiana* conidial suspension respectively, and the larvae dipped with sterile Tween 80 solution were set as controls, resulting in four separate treatment groups (Axenic; Nonaxenic; Bb + axenic; Bb + Nonaxenic). The larvae fed with the artificial medium/diet were kept at 26 °C and 90 ± 5% relative humidity. The number of surviving larvae was recorded daily.

### Microbiome analysis by 16S rRNA gene amplicon sequencing

Five days after infection, 10 gut and cuticle samples from each treatment of *M. alternatus* larvae challenged by Tween 80 and three levels of concentration (1 × 10^7^; 1 × 10^8^; 1 × 10^9^ conidia mL^−1^) Bb3275 were dissected (only live larvae were collected). All gut samples were processed with a DNA extraction kit (Fast DNA SPIN Kit for Soil) to extract the total community DNA. The quality and concentration of DNA were determined by 1.0% agarose gel electrophoresis and a NanoDrop® ND-2000 spectrophotometer (Thermo Scientific Inc., USA) and kept at − 80 °C prior to further use.

The DNA samples were sent to a sequencing company (Majorbio Bio-Pharm Technology Co. Ltd., Shanghai, China). 16s rRNA of distinct regions V3–V4 were amplified using universal primers 338F (5′-ACTCCTACGGGAGGCAGCAG-3′) and 806R (5′-GGACTACHVGGGTWTCTAAT-3′). The PCR reaction mixture including 4 μL 5 × Fast Pfu buffer, 2 μL 2.5 mM dNTPs, 0.8 μL each primer (5 μM), 0.4 μL Fast Pfu polymerase, 10 ng of template DNA, and ddH_2_O to a final volume of 20 µL. PCR amplification cycling conditions were as follows: 95 °C, 3 min; 27 cycles of 95 °C, 30 s; 55 °C, 30 s; 72 °C, 45 s; 72℃, 10 min (ABI GeneAmp 9700). PCR products were detected by 2% agarose gel electrophoresis.

### Analysis of bacterial microbiota

The raw 16S rRNA gene sequencing reads were demultiplexed and quality-filtered with Trimmomatic. Then, the paired-end (PE) reads were merged into a sequence using FLASH (V1.2.7) with a minimum overlap of 10 bp. Meanwhile, the quality of reads and the effect of merging were filtered, and the maximum error ratio allowed for overlapping area was 0.2. Samples were distinguished according to the barcode and primers, and the sequence direction was adjusted. The maximum number of barcodes was 0 and primer mismatches was 2, and the reads containing ambiguous characters were removed.

The optimized sequences were clustered into Operational Taxonomic Units (OTUs) with a similarity cutoff of 97% using Uparse (version 7.0.1090 http://drive5.com/uparse/). The OTUs were further subjected to a taxonomy-based analysis using the Ribosomal Database Project (RDP) algorithm (version 2.11 http://sourceforge.net/projects/rdp-classifier/) and confidence threshold set to 0.7. Silva (Release138 http://www.arb-silva.de) was used as an annotated database.

Alpha diversity using ACE, Chao1, Shannon and Simpson’s index as indicator of species richness and diversity, and beta diversity (weighted UniFrac, PCoA and NMDS) were analyzed by QIIME, then figures were made using R language (Version 3.3.1). Compositional differences in NMDS were tested using ANOSIM with 1000 permutations. A Permutational Multivariate Analysis of Variance based on the weighted UniFrac distance (PERMANOVA) was used to test for differences in PCoA between treatments.

### Quantification of total or specific bacteria

To confirm the variation of total bacteria and amounts of *Pseudomonas* and *Serratia* in the gut and on the cuticle of *M. alternatus* larvae after different treatments, a quantitative PCR approach was used. DNAs from gut and cuticle of the beetle larvae were extracted using TIANamp Genomic DNA kit (TIANGEN, Beijing, China). Then all samples were adjusted to the same concentration according to quantifications with a Nanodrop 2000. Quantitative Real-Time PCR was performed by a CFX96™ Real-Time System (Bio-Rad Laboratories, Hercules, CA, USA) in a volume of 20 μL using ChamQ Universal SYBR qPCR Master Mix (Vazyme, Nanjing, China) and the protocol was as follows: 95 °C for 30 s, then 40 cycles of 95 °C for 10 s, and 60 °C for 15 s. Data was analyzed using the 2^−ΔΔt^ method. The primer pair used for all bacteria was 16s-F (5′-TCCTACGGGAGGCAGCAGT-3′) and 16s-R (5′-GGACTACCAGGGTATCTAATCCTGTT-3′). The primer pair used for *Pseudomonas* quantification was Pse434F (5′-ACTTTAAGTTGGGAGGAAGGG-3′) and Pse686R (5′-ACACAGGAAATTCCACCACCC-3′), Sm-F2 (ACGTTCATCAATTGACGTTACTCGCA) and Sm-R2 (AACCGCCTGCGTGCGCTTTA) for *Serratia.* In addition, the larvae’s ActinF (5′-TGGGTATGGAATCTTGCGGT-3′) and ActinR (5′-GGCGGTGATTTCCTTTTGCA-3′) were used as reference genes.

### Identification of bacteria

*Monochamus alternatus* larvae associated bacterial isolates were identified by PCR amplification and sequencing of conserved 16S ribosomal RNAs. DNA was isolated using TIANamp Bacteria DNA Kit (TIANGEN, Beijing, China) as specified by the manufacturer for use as template in PCR. Nearly full length 16S rRNA was amplified for each isolate using primers universal to prokaryote 27F (5′-AGAGTTTGATCCTGGCTCAG-3′) and 1492R (5′-TACGGYTACCTTGTTACGACTT-3′). Thirty-five PCR cycles were conducted. The PCR final products were tested by electrophoresis at a 1% concentration of agarose gel, and band watched under UV reader.

DNA samples were sent to Sangon Biotech (Shanghai) Co., Ltd for sequencing. Then sequences of DNAs were edited and assembled (SeqMan component) and compared with those of known bacteria on NCBI-Genbank database. Finally, the phylogenetic tree was analyzed in Mega11 edition software (Additional file 1: Fig. S2).

### Antifungal activity assay

To determine the inhibitory effect of bacteria on fungal spore germination, *Pseudomonas protegens* (B1-S1-1L) and *Serratia marcescens* (C1-1-2L) were cultivated on LB (1% tryptone, 0.5% yeast extract, 0.5% NaCl) at 28 °C with shaking. The bacterium *Enterobacter soli* (C4-2-2L), isolated from *M. alternatus*, was incubated with the same method and set as a negative control. Adjusted to an optical density (OD) of 0.3 (OD_600_ = 0.3), we then inoculated the 10 μL 1:1 mixture of bacterial fluid and fungal spore suspension (10^5^ conidia mL^−1^) on PDA medium [24]. The plates were incubated for 12 h in the dark at 28 ± 1 °C, and germination was observed at 400× magnification [25]. A total of 100 conidia were scored for each treatment each time. Three independent repetitions of the experiment were performed.

To test antifungal activity, 20 μL bacterial fluid of each strain (OD_600_ = 0.3) was inoculated in 5 mm inoculation hole on 60 mm PDA medium which was initially coated with *B. bassiana* (10^7^ conidia mL^−1^) and the experiment was repeated three times for each treatment [18, 26]. The inhibition was measured after 3 days of incubation.

### qPCR analysis of immune-related genes and phenoloxidase activity assay

Since a similar antagonistic effect of *M. alternatus*-associated bacteria against *B. bassiana* was found among three dilutions of fungal treatments, we chose the concentration of *B. bassiana* spores at 1 × 10^7^ conidia mL^−1^ for further analysis. Five days after post-infection (dpi), the larval fat body samples from the four groups (Axenic; Nonaxenic; Bb + axenic; Bb + Nonaxenic) were taken; these were collected using a dissecting shear under aseptic conditions from five larvae and pooled as one sample (n = 3). All samples were stored at − 80 °C. Total RNA was extracted using RNA isolater Total RNA Extraction Reagent (Nanjing Vazyme Biotech Co, Ltd) following the manufacturer’s instructions. One μg RNA was reverse transcribed using HiScript® III RT SuperMix for qPCR (Vazyme, Nanjing, China). The q-PCR method was the same as described above. Each experiment was repeated three times independently. The primers are described in Additional file 1.

The hemocytes of axenic and nonaxenic *M. alternatus* larvae (14 larvae per group) were collected under aseptic conditions after 5 dpi. Then, using an insect peroxidase (PO) ELISA kit (JingMei Biotechnology, Jiangsu, China), we assayed the enzymatic reaction in different samples following the manufacturer’s instructions. Each sample and standard substance were placed in 96-well plates, at 37 °C for 60 min. Then, plates were washed five times by eluent for 30 s each, and ELISA HRP A, B was added and maintained at 37 °C for 15 min. The optical density value at 450 nm was measured after a stop reaction.

### Statistical analysis of data

Bacterial community composition was analyzed at phylum and genus levels, and species with an abundance ratio of less than 0.01 in all samples were classified as “other”. Kruskal–Wallis H-test was used to test for significant differences between groups among the top 10 genera according to their relative abundance.

Survival curves were analyzed using the Kaplan–Meier method, and the log-rank test was used to evaluate significant differences between two groups. As for other data, prior to analysis, we tested the residuals normality with the Shapiro–Wilk test for each level of variable. Data consisting of two treatments were analyzed using Student’s *t-*test and data consisting of more than two treatments were analyzed with one-way ANOVA coupled with a post hoc Waller–Duncan (equal variances) or Dunnett’s T3 (unequal variances) test. A value of *P* < 0.05 was considered significantly different. Data were analyzed using SPSS 19.0 and figures were made using GraphPad Prism 8.

## Results

### Axenic larvae of *M. alternatus* were more susceptible to fungal infection than non-axenic larvae

**Fig. 1:**
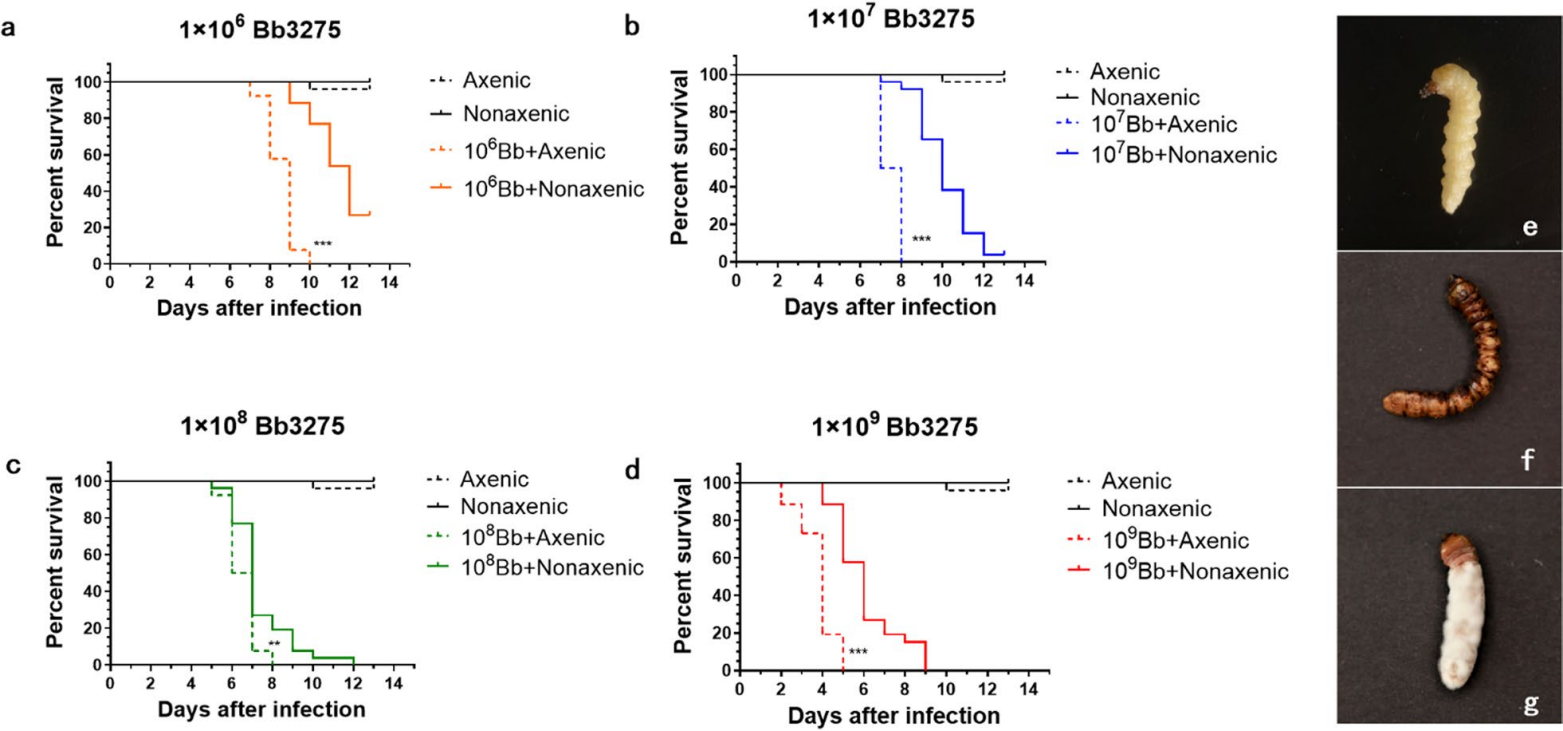
Survival of axenic and nonaxenic *Monochamus alternatus* larvae (n = 25) following topical infection with different concentration *Beauveria bassiana*. **a** 1 × 10^6^ conidia mL^−1^; **b** 1 × 10^7^ conidia mL^−1^; **c** 1 × 10^8^ conidia mL^−1^; **d** 1 × 10^9^ conidia mL^−1^ (Statistically significant differences between axenic and nonaxenic larvae infected Bb3275 are indicated by the asterisks). The phenotypes of naturally infected *M. alternatus* larvae, uninfected larvae (**e**). In the early stage of infection, the larva surface produced black spots (**f**). The cuticle of the larvae were covered with mycelium (**g**)

As a negative control, there was no significant difference in the survival rate between axenic and non-axenic *M. alternatus* larvae that were not exposed to *B. bassiana* (Fig. 1). Following inoculation with *B. bassiana*, the survival of axenic larvae was significantly lower than the survival of non-axenic larvae. After 10 dpi, all axenic *M. alternatus* larvae died, but the survival rate of non-axenic larvae was 76.92% at 1 × 10^6^ conidia mL^−1^ (Fig. 1a, Log-rank test, *P* < 0.001) and 38.46% at 1 × 10^7^ conidia mL^−1^ (Fig. 1b, Log-rank test, *P* < 0.001), respectively. The infection of *B. bassiana* on axenic larvae at concentrations of 1 × 10^8^ and 1 × 10^9^ conidia mL^−1^ resulted in 100% mortality after 8 dpi and 5 dpi (Fig. 1c, d). We observed melanization spots on the cuticle that later extended to the whole body, subsequently hardening the body and becoming covered by mycelium (Fig. 1f, g). Collectively, the associated microbiota may play a protective role when its host beetle *M. alternatus* is infected by entomopathogenic fungus *B. bassiana.*

### Infection with *B. bassiana* decreased richness and diversity of the gut and cuticle bacterial communities

After high throughput sequencing the V3–V4 of 16s rRNA genes, a total of 2,253,296 and 2,078,449 major sequences were obtained from 40 gut samples and 40 cuticle samples, respectively. Following the taxonomic assignment, a total of 860 OTUs were obtained in the gut samples, and 367 OTUs were identified in the cuticle samples at 97% sequence similarity.

In the gut samples, we found that bacterial species richness was dropped as indicated by Chao1 index, significantly decreasing after the infection of 1 × 10^8^ conidia mL^−1^ and 1 × 10^9^ conidia mL^−1^ *B. bassiana* spores (Table 1, Student’s *t* test, *P* < 0.05). Shannon and Simpson index changed slightly, indicating that change of bacterial species diversity in the gut was not significant after *B. bassiana* infection.

**Table 1:** Comparison of alpha diversity (Mean ± SD, n = 10) of *Monochamus alternatus* larvae

Treatment	Chao1		Shannon		Simpson	
	Gut	Cuticle	Gut	Cuticle	Gut	Cuticle
Tween-80	246.36 ± 46.51	92.71 ± 19.27	1.66 ± 0.36	2.10 ± 0.40	0.35 ± 0.11	0.20 ± 0.07
1 × 10^7^Bb	201.76 ± 79.63	89.17 ± 33.93	2.12 ± 0.62	1.63 ± 0.50*	0.22 ± 0.13*	0.32 ± 0.14*
1 × 10^8^Bb	140.54 ± 62.30***	133.53 ± 34.16**	1.67 ± 0.47	1.66 ± 0.54	0.33 ± 0.14	0.36 ± 0.18*
1 × 10^9^Bb	114.35 ± 31.59***	124.16 ± 39.15*	1.84 ± 0.61	1.81 ± 0.45	0.29 ± 0.20	0.28 ± 0.0.16

Gut and cuticle bacterial community separately compared between treatments with *Beauveria bassiana* and Tween-80 group

*Indicates significant differences, *P < 0.05, **P < 0.01, ***P < 0.001

However, fungal infection decreased the bacterial diversity on cuticle of *M. alternatus* larvae; the Shannon index (1.63 ± 0.50 vs. 2.10 ± 0.40, *P* = 0.03) after being infected with 1 × 10^7^ conidia mL^−1^ *B. bassiana* was significantly lower than the control group (smaller values of the Shannon index indicate lower species richness), and Simpson index (0.32 ± 0.14 vs. 0.20 ± 0.07, *P* = 0.03) in 1 × 10^7^ conidia mL^−1^ *B. bassiana* was significantly greater than the control group (higher values indicate greater dominance of the community by certain species) (Table 1, Student’s *t* test, *P* < 0.05). Variation in the Chao 1 index showed a similar trend with gut samples (Table1). These results illustrated that both microbiome richness and diversity of *M. alternatus* larvae were affected by pathogenic fungi.

**Fig. 2:**
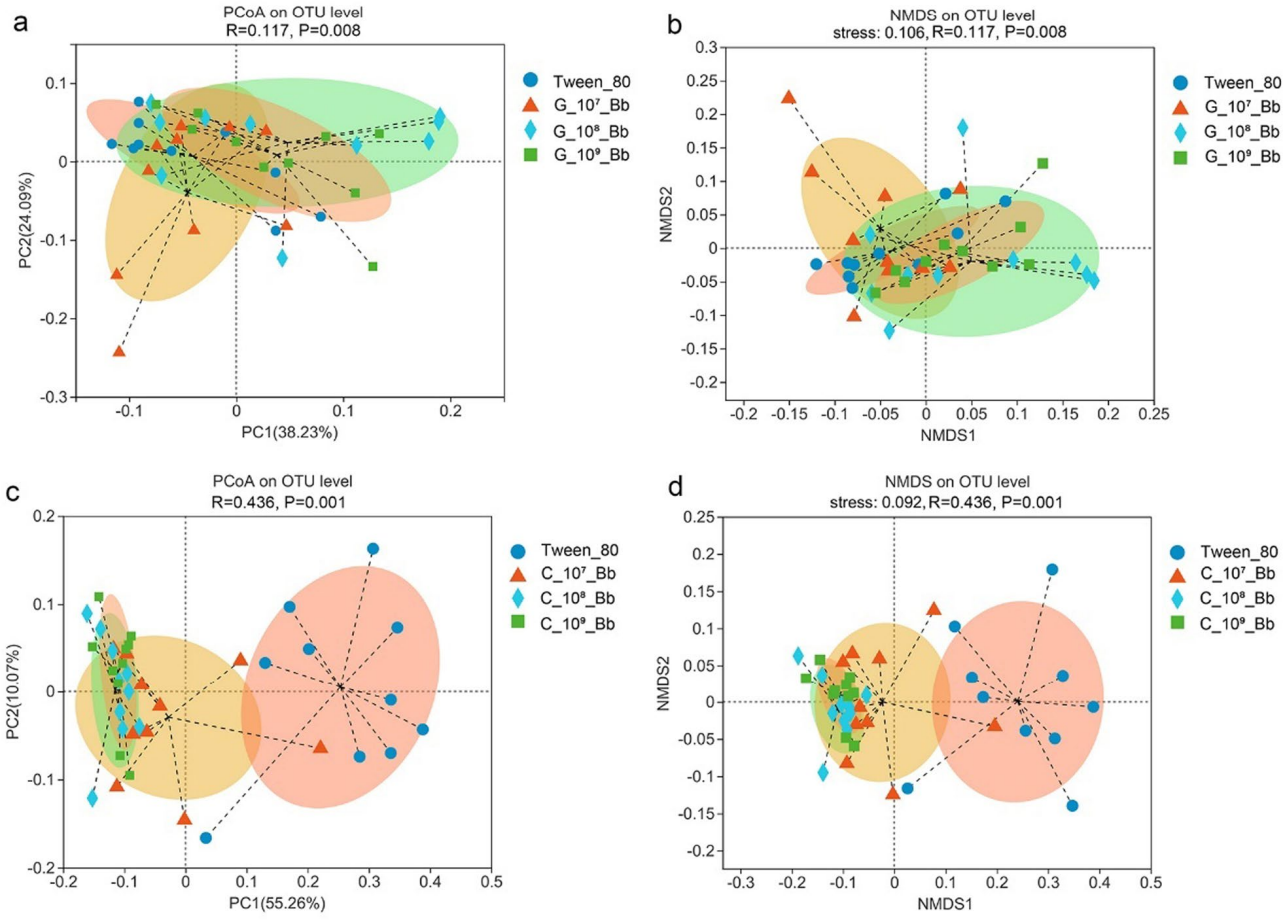
Principal coordinates analysis (PCoA) plots (**a**, **c**) and non-metric multidimensional scaling (NMDS) diagrams of associated bacteria structures (**b**, **d**) based on the OTU level used weighted UniFrac analysis. **a** and **b** Gut samples; **c** and **d** Cuticle samples

Principal coordinates analysis (PCoA) based on the weighted UniFrac analysis showed that gut and cuticle bacterial community structures of *M. alternatus* larvae were influenced differently after the treatments of *B. bassiana* (Fig. 2). In larval guts, bacteria in the pathogenic fungus-infected group were clustered together with those in untreated controls, with 38.23% and 24.09% of the variation explained by the principal components 1 and 2, respectively (Fig. 2a, b). Nevertheless, for *M. alternatus* larvae, the structure of the bacterial community residing on the cuticle surface changed notably following fungus inoculation. The bacterial communities in the ten control samples were very dispersive, but at each concentration of *B. bassiana* inoculation used, they were concentrated or aggregated in the PCoA plot (Fig. 2c). The analysis indicated that the infection of *B. bassiana* changed the cuticle bacterial community structure significantly (PERMANOVA, *P* < 0.01). Nonmetric multidimensional scaling (NMDS) based on weighted UniFrac analysis also followed a similar trend as PCoA (Fig. 2d, ANOSIM, *P* < 0.01).

### The abundance of several genera of bacteria increased after fungal infection

**Fig. 3:**
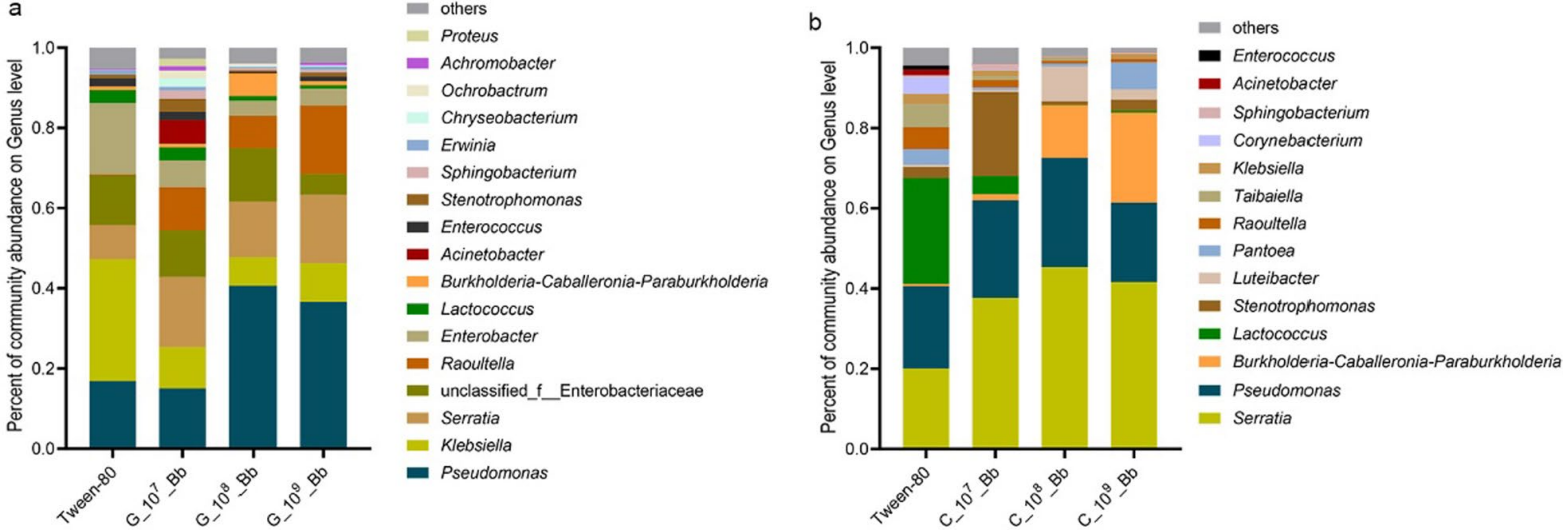
Bacterial community structure of different groups. Taxonomic classification of highly abundant (> 1%) members of *Monochamus alternatus* larvae in the guts and on the cuticle under different concentrations of *Beauveria bassiana*. Taxa with a abundance lower (< 1%) were summarized as “other”. **a** Gut; **b** Cuticle

**Fig. 4:**
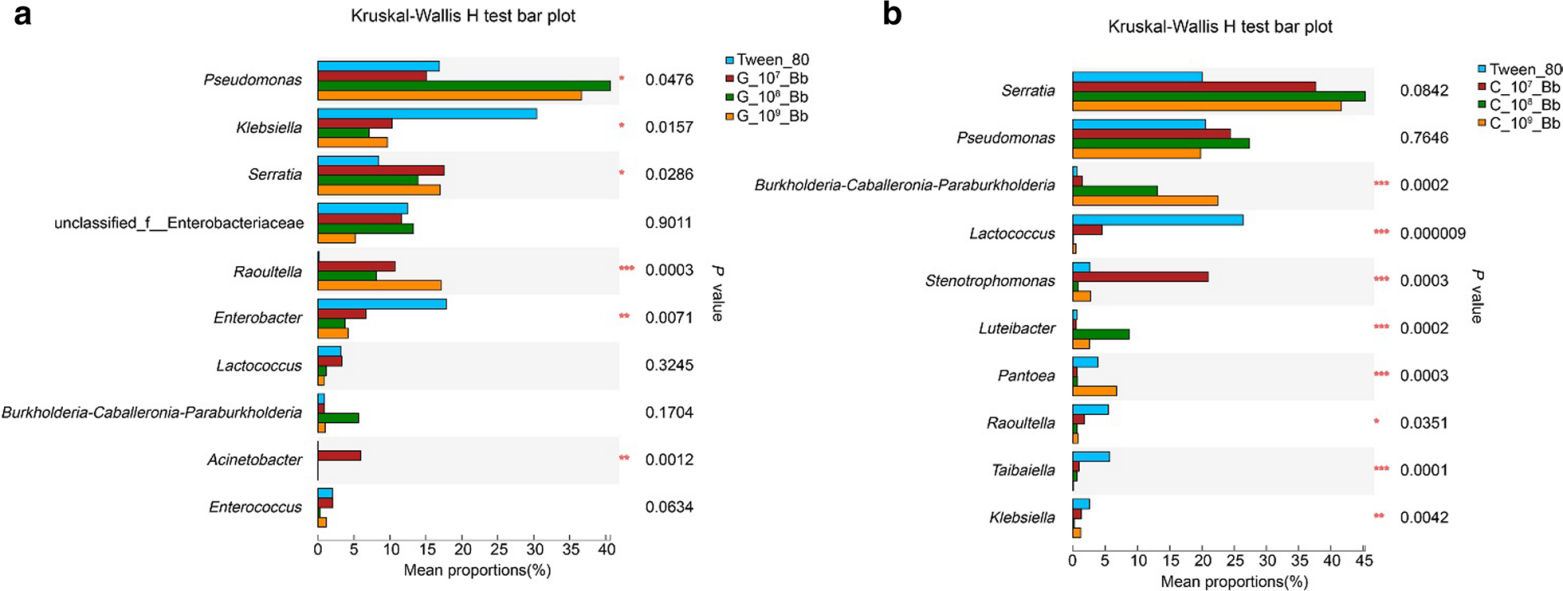
Significance analysis of differences among the top 10 genera in relative abundance from infected larvae and Tween treated larvae. **a** Gut; **b** Cuticle. *P < 0.05, **P < 0.01, ***P < 0.001

In terms of microbial composition, following inoculation with *B. bassiana,* the bacterial community composition in the guts and on the cuticle of *M. alternatus* larvae also changed, with *Pseudomona* sp. and *Serratia* sp. becoming the dominant bacteria (Fig. 3). Moreover, the abundance of *Pseudomona* sp. and *Serratia* sp. in the guts of *B. bassiana* infected larvae were significantly higher than those in control group (Fig. 4a, Kruskal–Wallis H-test, *P* < 0.05). Similar effects were obtained in the cuticle-associated bacteria of infected larvae (Fig. 4b, Kruskal–Wallis H-test, *P* < 0.05).

**Fig. 5:**
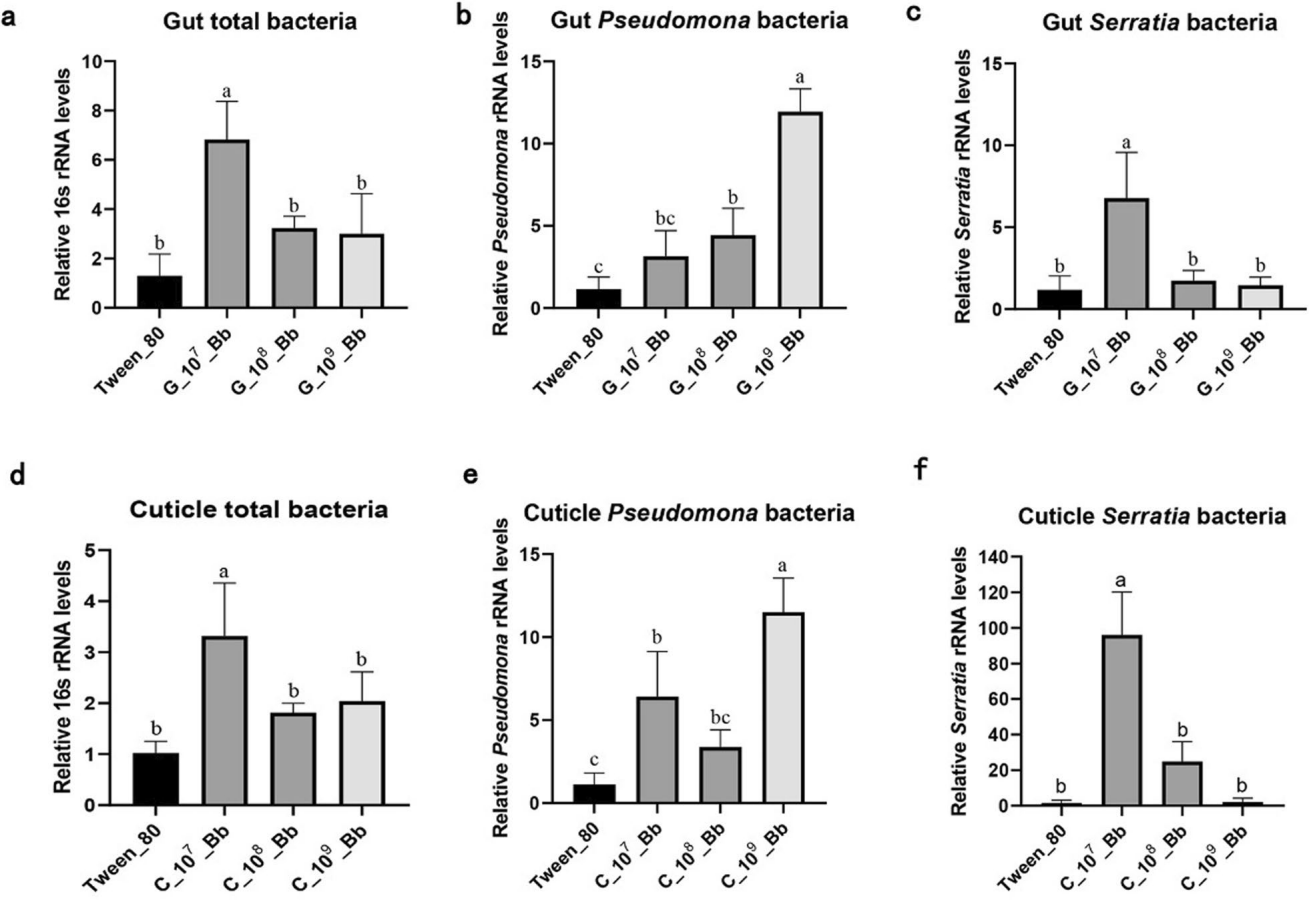
Number of gut and cuticle microbiota in *Monochamus alternatus* larvae infected by *Beauveria bassiana*. qPCR analysis on *M. alternatus* larvae (gut, n = 5; cuticle, n = 3) using universal 16S rRNA gene primers (**a**, **d**), *Pseudomonas* specific primers (**b**, **e**) and *Serratia* specific primers (**c**, **f**). Bars with different letters are significantly different (*P* < 0.05)

Our analyses also revealed a significant increase in the bacterial load caused by fungi. Specifically, the qPCR analysis showed that the total bacterial load in the guts and on the cuticle of the infected larvae at 1 × 10^7^ conidia mL^−1^ *B. bassiana* was significantly higher than the load in uninfected guts (Fig. 5a, d, ONE-WAY ANOVA, *P* < 0.05). Furthermore, qPCR analysis using *Pseudomona-*specific primers suggested that the *Pseudomona* sp. bacterial load in the infected group (1 × 10^7^–1 × 10^9^ conidia mL^−1^) at 5 dpi was much higher than in controls by several folds (Fig. 5b, e, ONE-WAY ANOVA, *P* < 0.01), and the higher infection concentration of *B. bassiana,* the greater the differences in the loads of *Pseudomona*. We also observed that the proportion of *Serratia* spp. increased both in the gut (6.78 ± 2.8 vs. 1.19 ± 0.85) and on the cuticle (96.02 ± 24.14 vs. 1.52 ± 1.62) of *M. alternatus* larvae after being inoculated at 1 × 10^7^ conidia mL^−1^ *B. bassiana* (Fig. 5c, f, ONE-WAY ANOVA, *P* < 0.01).

### The enriched bacteria showed high inhibitory effects on the fungal growth and spore germination

**Fig. 6:**
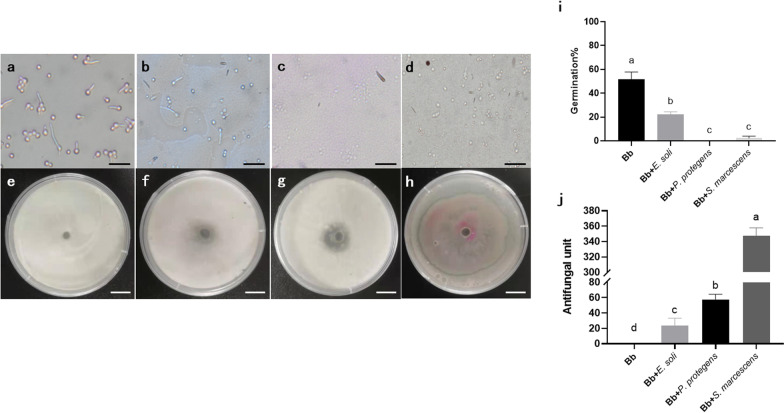
Germination of *Beauveria bassiana* was observed at × 400 magnification and in vitro assay of *Enterobacter soli* (**b**), *Pseudomonas protegens* (**c**) and *Serratia marcescens* (**d**) antifungal activity. Coculture bacteria of *E. soli* (**f**), *P. protegens* (**g**) and *S. marcescens* (**h**) and *B. bassiana* on PDA plates for 3 days. Inhibition was scored as the presence/absence of a distinct zone around inoculation hole. **a** and **e** showed the germination and culture of *B. bassiana* without bacteria. **i** Germination rate on PDA medium at 28 °C of *B. bassiana* germ tube under different bacteria coculture conditions and (**j**) bacteria antifungal units (= [inhibition diameter-5] × 10). Scale bars represent 20 μm (for **a**–**d**) and 10 mm (for **e**–**h**)

We evaluated whether the beetle associated bacteria were directly involved in resistance to pathogenic fungi. After 12 h incubation on PDA medium, the fungal germination rates were significantly different between treatment and control groups. The normal germination rate of Bb3275 at 12 h was 51.63 ± 6.21%. However, when it was co-cultured with gut bacteria of *M. alternatus* larvae, C4-2-2L (*Enterobacter* soil), B1-S1-1L (*Pseudomona protegens*) and C1-1-2L (*Serratia marcescens*), the fungal germination rates on PDA were significantly reduced down to 22.36 ± 2.0%, 0% and 2.40 ± 1.62%, respectively (Fig. 6i, ONE-WAY ANOVA, *P* < 0.001).

We also evaluated the diameter of bacterial inhibition zones (areas) on the PDA medium plates for calculating the bacteria antifungal units [= (inhibition diameter-5) × 10]. After 3 days, *B. bassiana* grew normally on the control plates without any bacterial inhibition zones. The average bacteria antifungal units on the medium inoculated with C4-2-2L, B1-S1-1L and C1-1-2L were 23.70 ± 9.46, 57.62 ± 6.74 and 347.68 ± 9.99, respectively (Fig. 6j, ONE-WAY ANOVA, *P* < 0.001).

### *Beauveria bassiana* provoked more intense immune responses to axenic larvae than non-axenic larvae

**Fig. 7:**
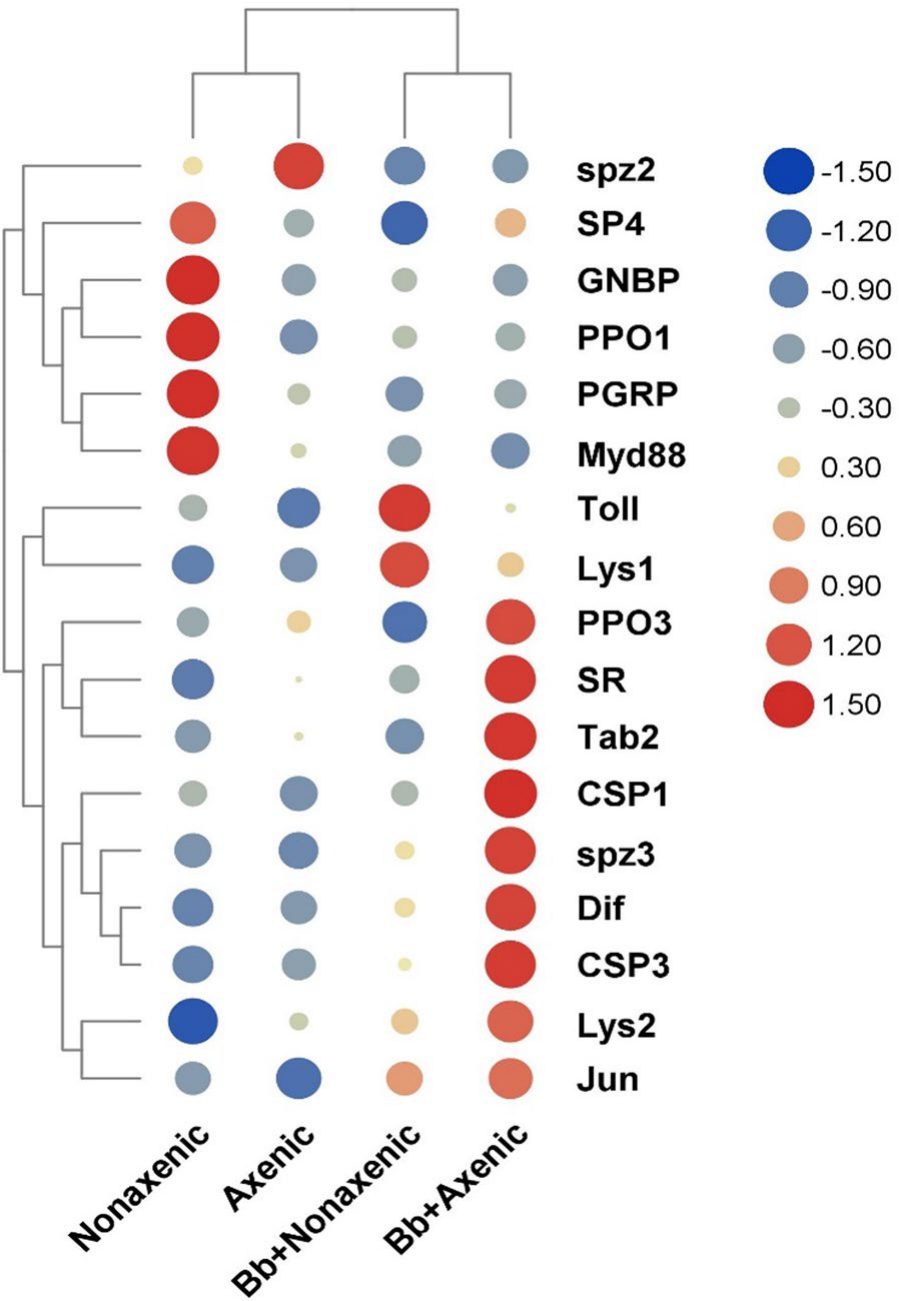
Differential responses of immunity-related genes in *Monochamus alternatus* following *Beauveria bassiana* infection among different treatment groups. Hierarchical clustering analysis of the main immunity related genes in nonaxenic and axenic *M. alternatus* larvae treated with Tween-80 or *B. bassiana*. Data represent mean of the Log2 fold change in expression. The size of dot and color shade indicates the Log2FC values. Gene names are shown on the right side

To better understand if beetle gut bacteria can enhance the expression of immune genes in the larvae of *M. alternatus*, we analyzed 17 immunity-related genes that were differentially expressed in recognition, signal modulation, the Toll pathway, IMD pathway, JNK pathway and effectors. At 5 dpi, recognition genes (PGRP, GNBP) were significantly down-regulated in axenic and non-axenic treatments. However, SR was up-regulated in axenic treatment. Interestingly, we observed signal modulation genes (CSP1, CSP3, SP4), Toll pathway (SPZ3, Dif), IMD pathway (Tab2) and JNK pathway (Jun) genes were both up-regulated in axenic *M. alternatus* larvae after being infected by the fungus. Moreover effectors (PPO3, Lys2) were down-regulated at 5 dpi in the fat body of non-axenic larvae (Fig. 7).

We further assayed PO enzymatic activity in the hemocyte of axenic and non-axenic larvae. We found that the PO content of axenic treatment was somewhat higher than conventional larvae though no statistic difference was detected (Additional file 1: Fig. S3). Combined with the results of qPCR, we showed that the infection of *B. bassiana* provoked higher immune responses to axenic larvae than to non-axenic larvae.

## Discussion

Our study revealed the role of *M. alternatus*-associated bacteria during entomopathogenic fungus infection. In the present work, by assessing the susceptibility of axenic larvae to four concentrations of *B. bassiana*, we suggest that the associated microbiome of *M. alternatus* is involved in the process of host resistance to *B. bassiana* infection. A similar effect was reported in the red palm weevil, in which the germ-free larvae died significantly faster than the conventionally reared larvae after being challenged by pathogenic bacteria, likely due to the fact that immunity-related genes were downregulated in germ-free larvae [27]. By using high-throughput sequencing and qPCR, we uncovered the dynamic change of associated bacteria of *M. alternatus*. Interestingly, the structure and richness of the beetle associated bacteria were profoundly changed after the fungal infection. It is worthy to note that the dominant bacterial species changed (or unchanged) and increased from treatment with *B. bassiana*. Species richness of core bacteria may be associated with host health [28]. Noskov et al. [29] observed that avermectins decreased the relative abundance of gut bacteria in *Aedes aegypti* and reduced the abundance of *Actinobacteria* with strong antifungal activity, which further caused an accelerated mortality of *A. aegypti* after fungal infection. Therefore, bacterial communities that differ in species richness or composition may vary in their defensive ability against pathogens. Nevertheless, in addition to bacteria, several Ophiostomatoid fungi were found to live on the cuticle of *M. alternatus* [30]. Thus, there is a possibility that the fungal species get involved in the antagonistic interaction with *B. bassiana* [31], which was not tested in the current study but deserves further investigations.

The bacterial communities of *M. alternatus* larvae are mostly represented by *Enterobacter*, *Raoultella*, *Lactococcus*, *Acinetobacter*, *Serratia*, *Pseudomonas*, and *Lactobacillus* at the genus level [22]. Our results are mostly consistent with earlier reports with regards to bacteria community composition (Fig. 3), however there were differences in the abundance of several species. In our study, the relative abundances of *Pseudomonas* and *Serratia* were the highest in conventional *M. alternatus* larvae*,* and in the guts and on the cuticle of the beetles, they were further enriched after *B. bassiana* infection (Fig. 3). Whether the fungal infection promoted these bacterial taxa in guts and on the cuticle together remains undetermined, because the horizontal transmission that occurs via contact or the fecal–oral route in insects enable gut bacterial colonization on the insect’s surface [32]. Either way, the increased abundance of bacteria has a potential to become involved as defensive intermediaries in the interaction between insects and their pathogens. For example, the bacteria associated with the integument of leaf-cutting ants could inhibit the fungal entomopathogen *Metarhizium anisopliae* [33]. It has been previously shown that *Pseudomonas* had chitinases [34] and *Serratia* was an effective bacterium for chitin degradation [35]. Thus, the elevation of *Pseudomonas* and *Serratia* of *M. alternatus* larvae may be caused by an increase in the fungal chitin content after *B. bassiana* infection.

In our current study, *Pseudomonas* and *Serratia* associated with *M. alternatus* larvae showed a strong inhibitory effect on the pathogenic fungi in vitro (Fig. 6). Similar results were found in other insects, for example, fungal growth was entirely suppressed by *Pseudomonas* sp. isolated from Colorado potato beetle [36]. Another study also showed that *P. reactans,* a bacterial strain isolated from the intestinal microbiota of *Blattella germanica*, significantly inhibited *B. bassiana* [37]. This inhibition of pathogenic fungi could be mainly attributed to the antibiotic secondary metabolites secreted by associated bacteria, which could directly inhibit the growth of entomopathogens. For instance, antifungal phenols produced by the gut bacteria of locusts could inhibit germination of *Metarhizium anisopliae* [38]. Antibiotic secondary metabolites that were secreted by *Actinobacteria* inhibit the growth of *Escovopsis* sp. [39]. Another reason behind the antagonistic effect between associated bacteria and fungi is nutritional competition as both agents live on the nutritionally limited resources [40]. The mechanism underlying the inhibition of *M. alternatus*-associated bacteria on *B. bassiana* remains unclear, and whether or not it is attributed to secreted antibiotic chemicals, nutritional competition, or other reasons, deserve further study.

On the other hand, we hypothesized that the presence of associated bacteria can help the host enhance humoral immunity. Previous studies have shown that immunity of insects could be induced by bacterial partners, and the enhanced expression of host immune-related genes and antimicrobial peptides further boost the host’s resistance to external aggressors or adversities [41–43]. For instance, the mealworm beetle (*Tenebrio molitor*), when treated by lipopolysaccharides (LPS), the component of some micro-organism cell walls, exhibited better survival following pathogen infection [44]. Clearly, bacterial stimulation of a host insect’s immune system is clearly not the case in *M. alternatus* as the expression quantity of immune-related genes in axenic larvae infected with *B. bassiana* had a more intense expression level than non-axenic larvae. In line with the expression pattern of immune genes, the PO activity in the hemocytes of axenic larvae is higher than those in non-axenic larvae before and after *B. bassiana* infection (Additional file 1: Fig. S3). Since a more intense innate immune response in an insect is likely to result in a more rapid energy depletion [45], the dependence of a bacterial partner which could directly inhibit pathogens is logistically superior for insects than the bacteria that stimulate or prime immunity to mitigate pathogenic infection. For example, in mosquitos, a similar phenomenon has been documented where mosquitos exposed to *Enterbacter* sp. bacterium isolated from wild mosquito populations could successfully eliminate infection of the pathogen *Plasmodium paraites.* The *Enterobacter* sp. bacterium could inhibit the development of *Plasmodium* regardless of the mosquito-derived innate immune response [46].

## Conclusions

In short, this study investigated the variation in the composition and structure of the bacterial community associated with *M. alternatus* larvae following infection with the biocontrol agent *B. bassiana.* We suggest that host insect’s defense system is linked to the shifts in the core bacterial composition. While the associated bacteria of *M. alternatus* inhibit the infection of pathogenic fungi, the underlying mechanisms remain unknown. Further studies, using metabolomics or proteomics are required to verify and elucidate the mechanisms of how bacterial partners shield the insect from its fungal pathogen. From the perspective of application, it is necessary to fully consider the protective effect of associated microorganisms of harmful insects to improve the effectiveness of biological control.

## Supplementary Information

**Additional file 1: Fig. S1.** Traditional isolating culturing *Monochamus alternatus *larvae gut (n = 3) homogenates on LB agar plates and TSA mediums indicated the efficiency of gut bacteria removal (**a**). Via universal 16S rRNA gene primers, we performed quantitative real-time reverse transcription polymerase chain reaction (qPCR) tested on *M. alternatus *larvae (n = 5) midgut homogenates (**b**). *P < 0.05. **Fig. S2.** Phylogenetic relationships among associated bacteria from *Monochamus alternatus* larvae and accepted type strains from their genera. Analysis showed that the 16s rRNA gene of C4-2-2L, B1-S1-1L and C1-1-2L isolate was clustered with *Enterobacter soli *ATCC BAA-2102 (98.92% percent identity), *Pseudomonas protegens* strain CHAO (100.00% percent identity), and *Serratia marcescens* strain NBRC (99.71% percent identity) respectively. **Fig. S3.** Phenoloxidase (PO) relative activity was evaluated from hemocyte of the *Monochamus alternatus *larvae. *Beauveria bassiana *infection results in PO activity increase in Axenic group. Bars with different letters are significantly different (*P* < 0.05).

## Data Availability

Not applicable.
