# Supplementary material for: Associated bacteria of a pine sawyer beetle confer resistance to entomopathogenic fungi via fungal growth inhibition
Source: Environ Microbiome. 2022 Sep 9;17:47. doi: 10.1186/s40793-022-00443-z (PMC9463743; doi:10.1186/s40793-022-00443-z)
Supplement: Supplementary file 1 — Additional file 1: Fig. S1. Traditional isolating culturing Monochamus alternatus larvae gut (n = 3) homogenates on LB agar plates and TSA mediums indicated the efficiency of gut bacteria removal (a). Via universal 16S rRNA gene primers, we performed quantitative real-time reverse transcription polymerase chain reaction (qPCR) tested on M. alternatus larvae (n = 5) midgut homogenates (b). *P < 0.05. Fig. S2. Phylogenetic relationships among associated bacteria from Monochamus alternatus larvae and accepted type strains from their genera. Analysis showed that the 16s rRNA gene of C4-2-2L, B1-S1-1L and C1-1-2L isolate was clustered with Enterobacter soli ATCC BAA-2102 (98.92% percent identity), Pseudomonas protegens strain CHAO (100.00% percent identity), and Serratia marcescens strain NBRC (99.71% percent identity) respectively. Fig. S3. Phenoloxidase (PO) relative activity was evaluated from hemocyte of the Monochamus alternatus larvae. Beauveria bassiana infection results in PO activity increase in Axenic group. Bars with different letters are significantly different (P < 0.05). [file 40793_2022_443_MOESM1_ESM.docx]

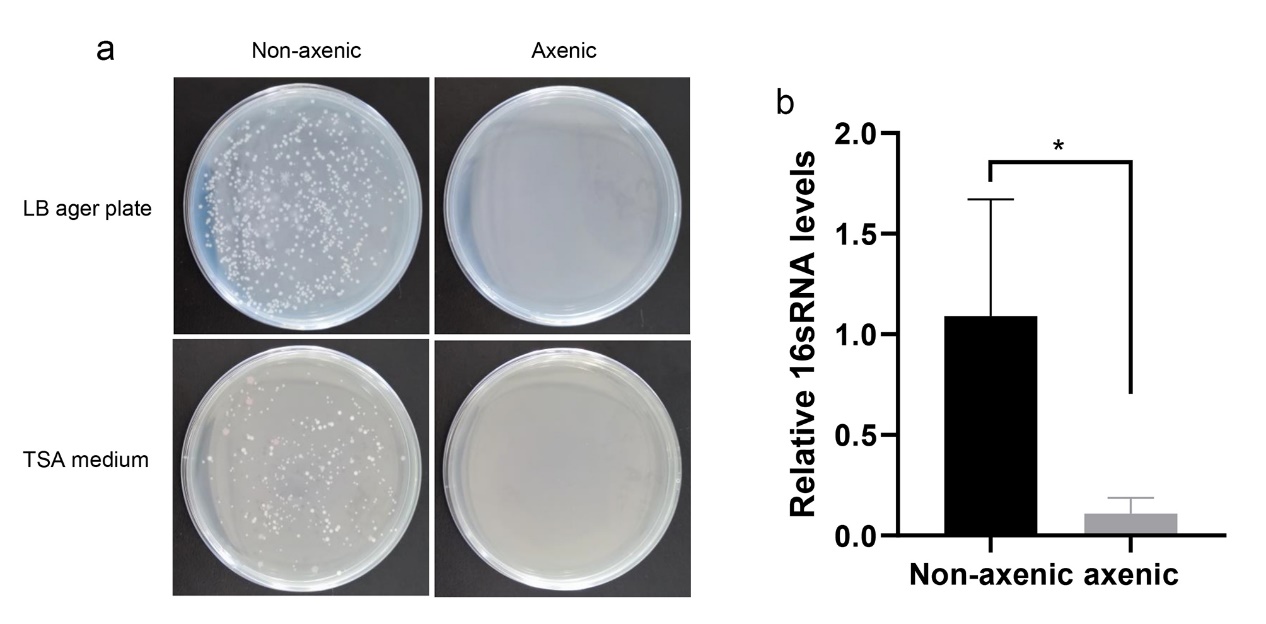
**Supporting information**

**Fig S1.** Traditional isolating culturing *Monochamus alternatus* larvae gut (n=3) homogenates on LB agar plates and TSA mediums indicated the efficiency of gut bacteria removal (a). Via universal 16S rRNA gene primers, we performed quantitative real-time reverse transcription polymerase chain reaction (qPCR) tested on *M. alternatus* larvae (n=5) midgut homogenates (b). **P* < 0.05.


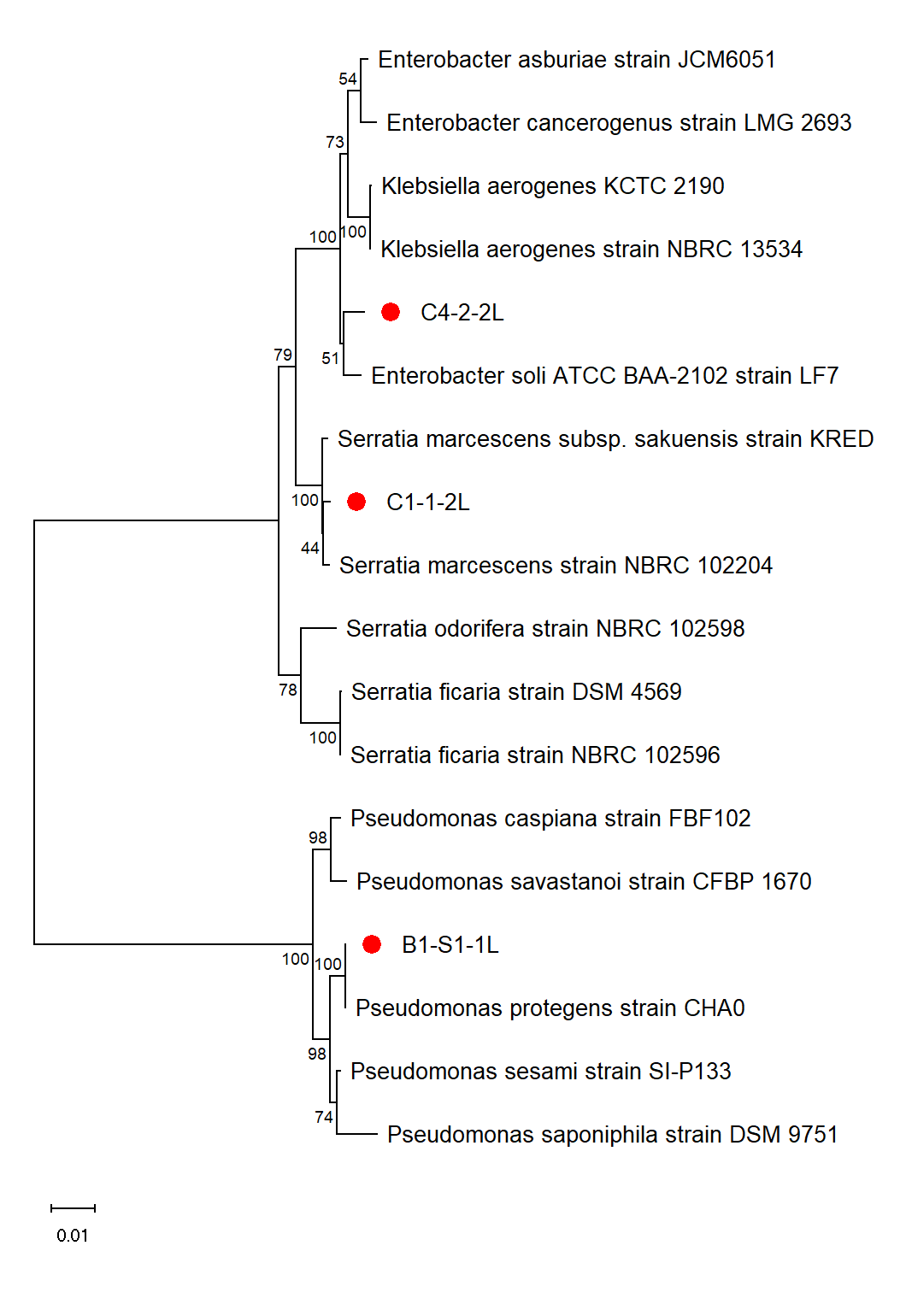


**Fig S2.** Phylogenetic relationships among associated bacteria from *Monochamus alternatus* larvae and accepted type strains from their genera. Analysis showed that the 16s rRNA gene of C4-2-2L, B1-S1-1L and C1-1-2L isolate was clustered with *Enterobacter soli* ATCC BAA-2102 (98.92% percent identity), *Pseudomonas protegens* strain CHAO (100.00% percent identity), and *Serratia marcescens* strain NBRC (99.71% percent identity) respectively.


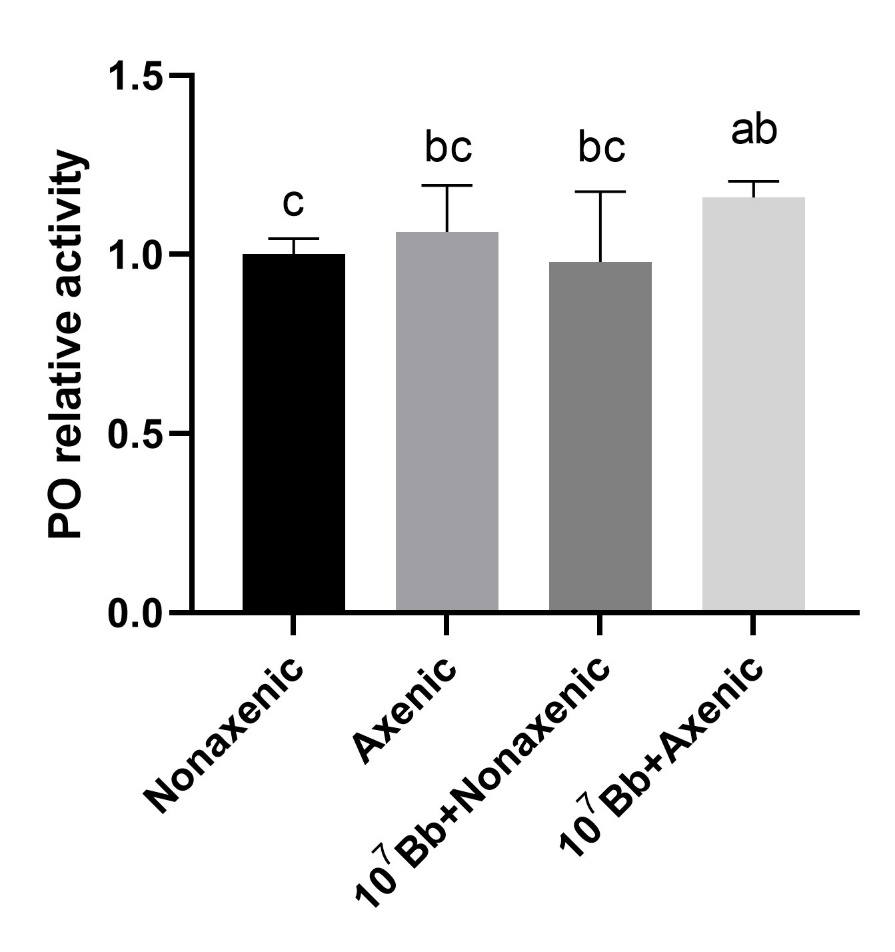


**Fig S3.** Phenoloxidase (PO) relative activity was evaluated from hemocyte of the *Monochamus alternatus* larvae. *Beauveria bassiana* infection results in PO activity increase in Axenic group. Bars with different letters are significantly different (*P*＜0.05).
